# The relationship of dietary fish intake to diabetic retinopathy and retinal vascular caliber in patients with type 2 diabetes

**DOI:** 10.1038/s41598-017-18930-6

**Published:** 2018-01-15

**Authors:** Jacqueline Chua, Ai-Ru Chia, Miao Li Chee, Ryan Eyn Kidd Man, Gavin Siew Wei Tan, Ecosse L. Lamoureux, Tien Yin Wong, Mary Foong-Fong Chong, Leopold Schmetterer

**Affiliations:** 1 Singapore Eye Research Institute, Singapore National Eye Centre, Singapore, Singapore; 2 Duke-NUS Medical School, Singapore, Singapore; 3 Department of Obstetrics & Gynecology, Yong Loo Lin School of Medicine, National University of Singapore, Singapore, Singapore; 4 Saw Swee Hock School of Public Health, National University of Singapore and National University Health System, Singapore, Singapore; 5 Singapore Institute for Clinical Sciences, Agency for Science, Technology and Research (A*STAR), Singapore, Singapore; 6 Clinical Nutrition Research Centre, Singapore Institute for Clinical Sciences, A*STAR, Singapore, Singapore; 7 Department of Ophthalmology, Lee Kong Chian School of Medicine, Nanyang Technological University, Singapore, Singapore; 8 Department of Clinical Pharmacology, Medical University of Vienna, Wien, Austria; 9 Center for Medical Physics and Biomedical Engineering, Medical University of Vienna, Wien, Austria

## Abstract

In this cross-sectional study, we evaluated the association of dietary fish intake with varying severity of diabetic retinopathy (DR) and retinal vascular caliber in Asians with type 2 diabetes mellitus. 357 Asians (median age: 58 years; 31% women; 78% Chinese) were recruited from a tertiary eye care institution in Singapore. Fish consumption was evaluated using a validated food frequency questionnaire. Digital retinal photographs assessed for DR severity and retinal vascular caliber. Ordered logistic and linear regression models were used to investigate the association of fish intake with DR severity and vascular caliber. Increasing frequency of fish consumption was significantly associated with lower odds of having severe DR (odds ratio [OR] = 0.91, 95% CI: 0.84–0.99 per 1-unit increase of fish intake; P = 0.038). Among those with no retinopathy, persons in quartile 4 fish intake had a wider retinal vascular caliber for arteriolar (β = 22.27 µm, 95% CI: 12.64–31.90; P-trend < 0.001) and venular (β = 32.00 µm, 95% CI: 17.56–46.43; P-trend < 0.001), than those in quartile 1 fish intake. Persons with higher fish intake had a decreased likelihood of having severe DR. In diabetics without retinopathy, higher fish intake was associated with wider retinal vascular caliber. Future research is needed to reinforce the direction of the casualty.

## Introduction

Diabetic retinopathy (DR) is the leading cause of visual impairment among working-age adults globally^1^. Studies have projected that the number of adults with type 2 diabetes mellitus in the world will rise further, with most of the increase occurring in Asia^2^. With the increasing prevalence of diabetes, the number of Asians affected by DR will increase^3^. As such, identifying effective strategies to prevent or delay diabetes-related visual impairment among Asians is a major public health challenge.

Recent studies have shown favorable associations between fish consumption and DR in several ethnic groups^4,5^. Sala-Vila *et al*. reported, at a 6-year follow-up, a relative decrease in risk for sight-threatening DR of 48% in Spanish adults with type 2 diabetes who consumed at least 500 mg/d of omega-3 fatty acids in their diet^5^. In an Australian study, patients with well-controlled diabetes, increasing daily intake of polyunsaturated fatty acids was associated with a reduced likelihood of DR^4^. In view of the concept that diet modification is one of the cornerstones of diabetes care, knowledge of such an association among Asians may provide clinically useful information for managing individuals with diabetes. Nevertheless, no such study has been performed in Asian populations, which possess different dietary patterns^6^, diabetes and DR burden^3^, compared with the Western population.

Furthermore, the exact mechanisms responsible for a protective effect of fish intake on DR remain unclear. To date several factors have been implicated in the pathophysiology of DR (e.g., vascular, metabolic and inflammatory)^7–9^, and determining if fish intake is related to early vascular pathways may provide insights into DR pathophysiology. To address this knowledge gap, we evaluated the association of dietary fish intake with the severity of DR in a well-characterized sample of Asian individuals with type 2 diabetes In Singapore. We further explored the relationship between fish intake and retinal vascular caliber to determine if fish consumption is linked with retinal microvasculature. We hypothesized that Asian individuals with higher fish intake will have less severe DR, with differences in retinal vascular diameters.

## Methods

### Study Population

**Figure 1:**
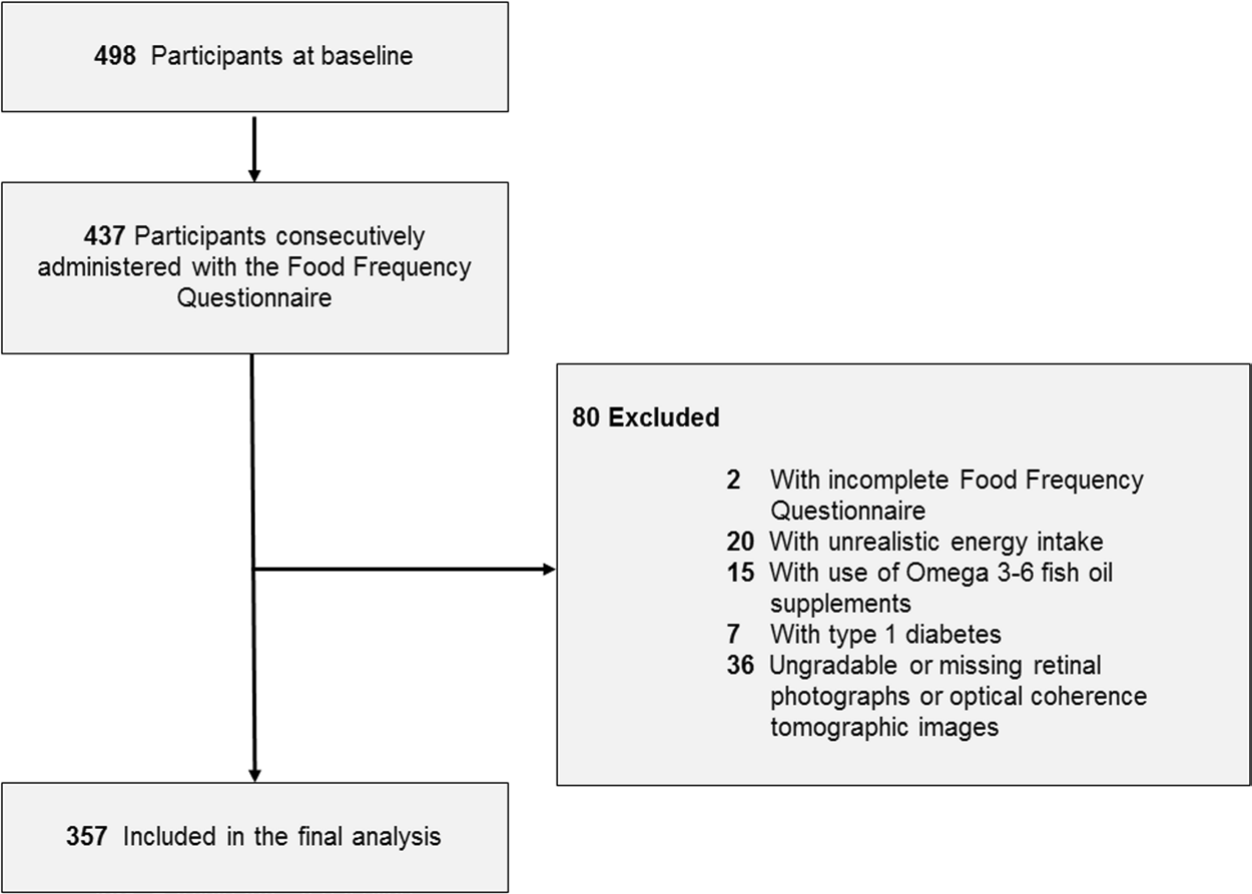
Identification of Eligible Participants from the Singapore Diabetes Management Project.

The Singapore Diabetes Management Project (S-DMP) is a clinical cross-sectional study investigating the clinical, behavioral, and environmental barriers associated with optimal diabetes care in individuals with diabetes with and without DR. Details of the study design and methodology has been described previously^10,11^. Briefly, 498 individuals with types 1 and 2 diabetes, aged 21 years and older, of Asian ethnicity (Chinese, Malay, and Indian), were recruited from the Singapore National Eye Centre, a tertiary eye care institution in Singapore, from December 2010 to September 2013. All participants were free from cognitive impairment (assessed using the 6-item Cognitive Impairment Test)^12^, were of sufficient hearing to be able to conduct normal conversations, and lived independently in the community (i.e., not living in assisted care facilities). Presence of diabetes was defined as physician diagnosed diabetes, with the information retrieved from participants’ case notes. Approval for conducting this study, including the consent procedure, was obtained from the Singapore Centralized Institutional Review Board (reference 2010/470/A), and all study procedures adhered to recommendations of the Declaration of Helsinki. Written Informed consent was obtained from all participants. Of the S-DMP participants, 437 consecutive participants were administered the Food Frequency Questionnaire (FFQ) (see later section). For this study, we excluded participants with incomplete FFQ (n = 2), unrealistic energy intake (n = 20), use of omega 3–6 fish oil supplements (n = 15), type 1 diabetes (n = 7) and ungradable or missing retinal photographs or optical coherence tomographic (OCT) images (n = 36), leaving 357 participants for analysis (Fig. 1).

### Assessment of Diabetic Retinopathy and Vision-threatening Diabetic Retinopathy

Digital retinal photography was performed using a 45° retinal camera (Canon CR6–45NM; Canon, Inc., Tokyo, Japan) after pharmacological pupil dilation. Two retinal images of each eye were obtained, one centered at the optic disc and another centered at the fovea. Trained graders, masked to participant characteristics, assessed for DR severity and measurement of retinal vascular caliber.

Severity of DR was graded from the 2-field digital retinal photographs using the modified Airlie House classification system^13^. Diabetic macular edema was defined by hard exudates in the presence of microaneurysms and blot hemorrhage within 1 disc diameter from the foveal center or the presence of focal photocoagulation scars in the macular area^14^. Clinically significant macular edema (CSME) was considered present when the macular edema was within 500 µm of the foveal center or if focal laser photocoagulation scars were present in the macular area. Diabetic laser treatment was defined based on presence of laser focal/scatter photocoagulation scars. These were confirmed with central macular thickness measurements by optical coherence tomography (OCT; Cirrus Version 3.0; Carl Zeiss Meditec) using the macular thickness cube scan protocol (512 × 128). Only scans with signal strength ≥6 were included. Central macular thickness was defined as the central circular zone of 1 mm in the scan. We defined two primary outcomes for this study based on the severity scores of the worse eye. DR was divided into no retinopathy present (Early Treatment Diabetic Retinopathy Study [ETDRS] levels 10–15), minimal-moderate non-proliferative DR (NPDR; levels 20–47), and severe NPDR or worse (levels ≥ 53). Vision-threatening DR (VTDR) was defined as the presence of severe NPDR-proliferative retinopathy or CSME. If an eye was ungradable, the score for the other eye was used to define these outcomes. The participant was excluded from analysis if images from both eyes were ungradable.

### Measurement of Retinal Vascular Caliber

Retinal vascular caliber was measured from retinal photograph, with a semi-automated computer-assisted program (Singapore I Vessel Assessment [SIVA], version 3.0)^15^. Optic disc-centered photograph of the right eye of each participant were selected for measurement; if the right eye photograph was ungradable, measurements were performed on the left eye. For each photograph, all arterioles and venules coursing through an area 0.5 to 2 disc diameter from the optic disc margin were measured and summarized as the average central arteriolar and venular equivalents^16^. These equivalents are projected calibers for the central retinal vessels, measured away from the optic disc and had a high intergrader repeatability (intraclass correlation coefficients > 0.90)^17^.

### Dietary Assessment

Dietary intake over the preceding month was assessed using a validated 172-item semiquantitative food frequency questionnaires (FFQ), modified for the Singapore diet^18^. The FFQ was administered by trained interviewers and participants were asked to estimate their frequency (per day, per week, per month or never/rarely) of consuming each food item based on a standard portion size. Visual aids such as standardized household measuring utensils and food pictures were provided to assist participants in quantifying their food intake. Nutrient values were primarily obtained from a food composition database of locally available foods, with modifications made on inaccuracies found^19^. For food items not found in the database, nutrient information was obtained from US^20^, UK^21^, or Australia^22^ food composition database. The average daily nutrient intake for each participant was calculated by multiplying the frequency of consumption of each item by its nutrient content of the portion size specified on the FFQ and summing the nutrient intake for all food items. Participants were defined as having unrealistic energy intake if they reported total energy intakes outside predefined limits (<800 or >4200 kcal/day for men, and <500 or >3500 kcal/d for women)^23^. Data on fish intake were obtained by totaling the frequency of consumption of a standard serving of fish (90 grams) in seven items accounting for different cooking methods – stir-fried/pan-fried/deep fried, deep fried with batter, steamed, assam pedas (spicy and sour tamarind-flavored fish), coconut curry, curry without coconut and grilled.

### Assessment of Covariates

Information on participants’ demographic and socioeconomic status (e.g. education and income), smoking status, duration of diabetes, and medication use were collected using a standardized questionnaire. Participants were also asked the type of diabetic treatment they were currently using (diet alone, oral agents i.e. anti-hyperglycemic or insulin). Body mass index (BMI) was calculated as body weight (in kilograms) divided by body height (in meters) squared. Seated blood pressure (BP) was performed using a digital blood pressure oscillometer (Dinamap Pro 100 V2; GE Heathcare). Nonfasting venous blood samples were also collected to assess glycated hemoglobin (HbA1c), serum total cholesterol, high-density lipoprotein cholesterol (HDL), low-density lipoprotein cholesterol (LDL), and triglyceride levels. Total cholesterol to HDL cholesterol ratio was used as a measure of dyslipidemia in analyses because this measure has greater predictive value in terms of physiological and clinical significance than isolated parameters used independently^24^. Hypertension was defined as systolic BP ≥ 140 mmHg, and/or diastolic BP ≥ 90 mmHg, and/or history of antihypertensive medication. Dyslipidemia was defined as a total cholesterol level ≥ 6.2 mmol/L and/or history of lipid-lowering drugs.

### Statistical Analysis

Baseline characteristics are presented for overall sample and also for subgroups stratified by quartiles of fish intake. The Shapiro-Wilk test was used to assess the normality of the distribution of the variables. Continuous variables that are normally distributed are presented as mean (standard deviation) whereas non-normally distributed variables are presented as median (interquartile range [IQR]). P-trends were assessed by modeling the mean value of the quartiles in the linear regression analysis (normally distributed continuous), median value of the quartiles in a linear regression analysis (non-normally distributed continuous) or with the use of the chi-square test for linear trends (categorical). The proportional odds assumption between each level of DR severity was assessed with the likelihood ratio test. Multivariable ordered logistic and linear regression models were used to investigate the association of fish intake (exposure) with DR severity and vascular caliber (outcomes), respectively, adjusted for potential confounding factors. We adjusted for variables such as age, gender, race, smoking, diabetes duration, insulin use, lipid-lowering medication use, BMI, systolic blood pressure, HbA1c, triglyceride level, and total to HDL cholesterol ratio in the multivariate model if they had a statistical significance of P value < 0.10 in Model 1 (age, gender and race adjusted). We also measured for multicollinearity using the variance inflation factor (VIF), which assessed how much variances of an estimated regression coefficient increases if our predictors are correlated. Our results indicate the all VIFs were less than 2, indicating that there is no multicollinearity in our multivariate regression analyses. Data were analyzed with Stata 13 (StataCorp, Texas, USA).

## Results

### Characteristics of the Study Participants

The characteristics of the 357 participants included in the study are summarized according to quartiles of fish intake (Table 1). The median age and interquartile range [IQR] of these participants was 58 years (IQR: 52–62), 31% were female (n = 110), and 78% were of Chinese ethnicity (n = 279). The median duration of diabetes was 11 years (IQR: 5–20) and HbA1c level was 7.3% (IQR: 6.7–8.5). On average, the median fish intake was 3 times (3 servings) per week (IQR: 1–4). The overall prevalence of any DR was 60%, consisting of 141 (40%) with minimal-moderate NPDR, and 75 (20%) with severe NPDR or worse. 86 (24%) had VTDR. The median arteriolar diameter in this sample was 132 μm (IQR: 123–140) whereas the venular diameter was 194 μm (IQR: 183–210). Persons with a higher fish intake had a lower total to HDL cholesterol ratio, higher total energy intake, higher intakes of vegetables and fruits and lower intakes of meat (all P-trends < 0.05).

**Table 1:** Participants’ sociodemographic and clinical characteristics stratified by fish intake.

Characteristics	Total	Fish intake category (grams of fish per week)				P-trend^*^
		Quartile 1 (22.5–90 grams)	Quartile 2 (180–270 grams)	Quartile 3 (360–360 grams)	Quartile 4 (450–630 grams)	
N, %	357 (100%)	98 (28%)	82 (23%)	101 (28%)	76 (21%)	
Age, years	58 (52, 62)	56 (51, 61)	59 (53, 62)	58 (54, 61)	58 (54, 63)	0.169
Gender, %						
Male	247 (69%)	64 (65%)	58 (71%)	71 (70%)	54 (71%)	0.422
Female	110 (31%)	34 (35%)	24 (29%)	30 (30%)	22 (29%)	
Ethnicity, %						
Chinese	279 (78%)	75 (77%)	72 (88%)	71 (70%)	61 (80%)	0.899
Malay	26 (7%)	4 (4%)	4 (5%)	13 (13%)	5 (7%)	
Indian	46 (13%)	17 (17%)	5 (6%)	15 (15%)	9 (12%)	
Others	6 (2%)	2 (2%)	1 (1%)	2 (2%)	1 (1%)	
Education, %						
Low (6 years or less)	96 (27%)	24 (24%)	24 (29%)	30 (30%)	18 (24%)	0.959
High (more than 6 years)	260 (73%)	74 (76%)	58 (71%)	70 (70%)	58 (76%)	
Income, %						
Low (less than $2000/month)	110 (37%)	33 (38%)	29 (45%)	30 (37%)	18 (26%)	0.106
High ($2000/month or more)	191 (63%)	53 (62%)	36 (55%)	52 (63%)	50 (74%)	
Duration of diabetes, years	11 (5, 20)	12 (5, 20)	11 (5, 20)	10 (6, 16)	13 (5, 21)	1.000
Current smoker, %	35 (10%)	11 (11%)	9 (11%)	8 (8%)	7 (9%)	0.518
Diabetic treatment						
Diet alone, %	182 (51%)	47 (48%)	46 (56%)	51 (51%)	38 (50%)	0.793
Oral agents, %	163 (46%)	47 (48%)	32 (39%)	47 (47%)	37 (49%)	
Taking insulin, %	11 (3%)	3 (4%)	4 (5%)	3 (3%)	1 (1%)	
Taking lipid-lowering medication, %	128 (36%)	35 (36%)	30 (37%)	38 (38%)	25 (33%)	0.758
Taking antihypertensive medication, %	124 (35%)	38 (39%)	27 (33%)	34 (34%)	25 (33%)	0.432
Body mass index (kg/m^2^)	25.8 (23.5, 29.3)	25.7 (22.8, 29.3)	25.9 (23.6, 28.5)	25.6 (23.9, 29.4)	25.8 (23.8, 30.2)	0.897
Systolic blood pressure (mmHg)	136.7 (17.4)	134.9 (19.5)	136.7 (17.6)	137.3 (14.7)	138.7 (15.5)	0.165
Diastolic blood pressure (mmHg)	77.0 (70.5, 84.5)	75.8 (70.5, 83.5)	75.5 (70.0, 84.5)	79.0 (73.5, 84.0)	78.5 (70.8, 85.3)	0.095
HbA1c (%)	7.3 (6.7, 8.5)	7.4 (6.8, 8.6)	7.2 (6.6, 8.4)	7.2 (6.8, 8.7)	7.4 (6.8, 8.8)	0.701
Triglycerides (mmol/l)	1.9 (1.3, 2.7)	2.0 (1.3, 3.2)	1.9 (1.3, 2.7)	2.1 (1.6, 2.9)	1.8 (1.3, 2.4)	0.444
Total to HDL cholesterol ratio	4.0 (3.4, 4.9)	4.3 (3.6, 5.0)	4.0 (3.2, 4.9)	4.0 (3.4, 4.5)	3.8 (3.3, 4.8)	0.013
Hypertension, %	207 (58%)	59 (60%)	45 (55%)	60 (59%)	43 (57%)	0.781
Dyslipidemia, %	128 (36%)	35 (36%)	30 (37%)	38 (38%)	25 (33%)	0.801
Nutrient intake						
Total energy intake, kcal/d	1826 (1414, 2390)	1616 (1356, 1959)	1834 (1389, 2321)	1947 (1682, 2456)	2042 (1540, 2657)	0.001
Rice/Noodle/Bread intake, no. of times per week	26 (19, 34)	24 (17, 33)	26 (19, 33)	27 (20, 35)	27 (21, 35)	0.151
Vegetable/Fruit intake, no. of times per week	21 (14, 34)	18 (10, 25)	20 (14, 35)	26 (19, 39)	24 (16, 37)	0.001
Meat intake, no. of times per week	4 (2, 7)	3 (1, 5)	4 (2, 7)	5 (4, 7)	5 (2, 8)	<0.001
Fish intake, no. of times per week	3 (1, 4)	1 (0, 1)	2 (2, 3)	4 (4, 4)	6 (5, 7)	<0.001
Severity of diabetic retinopathy, %						
No retinopathy	141 (40%)	36 (37%)	28 (34%)	48 (48%)	29 (38%)	0.177
Minimal-Moderate diabetic retinopathy	141 (40%)	37 (38%)	36 (44%)	34 (34%)	34 (45%)	
Severe diabetic retinopathy or worse	75 (20%)	25 (26%)	18 (22%)	19 (18%)	13 (17%)	
Vision-threatening diabetic retinopathy, %	86 (24%)	27 (28%)	20 (24%)	24 (24%)	15 (20%)	0.243
Diabetic retinopathy laser treatment						
No	292 (82%)	76 (78%)	64 (78%)	82 (81%)	70 (92%)	0.017
Yes	65 (18%)	22 (22%)	18 (22%)	19 (19%)	6 (8%)	
Retinal Vascular Caliber						
Arteriolar caliber, μm	132 (123, 140)	130 (121, 140)	133 (122, 142)	132 (125, 139)	134 (125, 143)	0.150
Venular caliber, μm	194 (183, 210)	193 (177, 217)	194 (183, 211)	191 (184, 204)	197 (187, 208)	0.376

HbA1c = hemoglobin A1C; HDL = high-density lipoprotein.

*P-trends were assessed by modeling the mean value of the quartiles in the linear regression analysis (normally distributed continuous), median value of the quartiles in the linear regression analysis (non-normally distributed continuous) or with the use of the chi-square test for linear trends (categorical).

Data presented are mean (standard deviation) for normally distributed variables, median (interquartile range) for non-normally distributed variables or number of participants (%) for categorical variables.

### Associations between Fish intake and Severity of DR

Table 2 shows the association between fish intake and the severity of DR using an ordered logistic regression analysis. After adjusting for age, gender, and race, dietary fish intake was inversely associated with varying severity of DR (odds ratio [OR], 0.39; 95% confidence interval (CI): 0.20 to 0.77; P = 0.007, per categorical increase in severity; Model 1 in Table 2), comparing those in quartile 3 versus quartile 1. In the multivariable-adjusted model, those in quartile 3 had lower odds of severe DR or worse versus the combined no DR and minimal-moderate DR categories, than those in quartile 1 (OR, 0.35; 95% CI: 0.17 to 0.74; P = 0.006; per categorical increase in severity Model 2 in Table 2), and a significant trend for increasing frequency of fish consumption (P-trend = 0.024). We found that persons with a higher fish intake were significantly associated with presence of severe DR (OR, 0.91 for one serving increase of fish intake per week, P = 0.038, Model 2 in Table 2), suggesting that higher serving of fish intake had a lower odd of severe DR occurrence compared with no DR. That is, having one serving more of fish intake per week reduced the likelihood of severe DR by almost 10%.

**Table 2:** Association between fish intake and severity of diabetic retinopathy in multivariable ordered logistic regression models.

	Model 1		Model 2	
	OR (95% CI)	P value	OR (95% CI)	P value
Fish intake category				
Quartile 1	1 (Reference)		1 (Reference)	
Quartile 2	0.89 (0.56 to 1.43)	0.638	0.79 (0.47 to 1.32)	0.367
Quartile 3	0.39 (0.20 to 0.77)	0.007	0.35 (0.17 to 0.74)	0.006
Quartile 4	0.72 (0.42 to 1.24)	0.239	0.58 (0.32 to 1.06)	0.076
P-trend^*^	0.080		0.024	
Per one serving increase of fish intake per week	0.94 (0.88 to 0.99)	0.038	0.91 (0.84 to 0.99)	0.038

OR, odds ratio; CI = confidence interval; DR = diabetic retinopathy.

*P-value to test for differences in trend between categories of fish intake (quartiles).

Fish intake, either as a categorical and continuous variable, was included one at a time in the multivariable ordered logistic regression models for DR Severity (no retinopathy present, minimal-moderate non-proliferative DR, and severe DR or worse.

Model 1 adjusted for age, gender and race.

Model 2 adjusted for age, gender, race, and variables with P < 0.10 in model 1 were included (smoking, diabetes duration, diabetic treatment, lipid-lowering medication use, systolic blood pressure, HbA1c and triglycerides levels).

We also performed a sensitivity analysis for Table 2 by including participants who took omega 3–6 fish oil supplements. Our new results (OR, 0.34; 95% CI: 0.16 to 0.71; P = 0.004; per categorical increase in severity; fully adjusted) indicate that the earlier associations between dietary fish intake and DR remained statistically significant and consistent in the direction as seen in Table 2.

### Associations between Fish intake and VTDR

Association between fish intake and VTDR (Table 3) was largely similar to the results presented for DR. Persons in quartile 3 had a significantly lower odds of having VTDR versus no VTDR, than for those in quartile 1 (OR, 0.38; 95%CI: 0.18 to 0.80; P = 0.010; Model 2 in Table 3), and a significant trend for increasing frequency of fish consumption (P-trend = 0.026). Frequency of fish consumption was also significantly associated with severity of VTDR in the multivariable-adjusted model (OR = 0.91, 95% CI: 0.84 to 0.99 per one serving increase of fish intake per week; P = 0.035; Model 2 in Table 3). There was no association between dietary fish intake and severe DR (Table 2) or VTDR (Table 3) when comparing quartile 4 to quartile 1.

**Table 3:** Association between fish intake and vision-threatening diabetic retinopathy in multivariable ordered logistic regression models.

	Model 1		Model 2	
	OR (95% CI)	P value	OR (95% CI)	P value
Fish intake category				
Quartile 1	1 (Reference)		1 (Reference)	
Quartile 2	0.94 (0.59 to 1.51)	0.807	0.87 (0.53 to 1.45)	0.605
Quartile 3	0.40 (0.20 to 0.80)	0.009	0.38 (0.18 to 0.80)	0.010
Quartile 4	0.74 (0.43 to 1.27)	0.272	0.60 (0.33 to 1.08)	0.089
P-trend^*^	0.089		0.026	
Per one serving increase of fish intake per week	0.93 (0.88 to 0.99)	0.036	0.91 (0.84 to 0.99)	0.035

OR, odds ratio; CI = confidence interval; DR = diabetic retinopathy.

*P-value to test for differences in trend between categories of fish intake (quartiles).

Fish intake, either as a categorical and continuous variable, was included one at a time in the multivariable ordered logistic regression models for DR Severity (no retinopathy present, minimal-moderate non-proliferative DR, and vision-threatening DR.

Model 1 adjusted for age, gender and race.

Model 2 adjusted for age, gender, race, and variables with P < 0.10 in model 1 were included (smoking, diabetes duration, diabetic treatment, lipid-lowering medication use, systolic blood pressure, HbA1c and triglycerides levels).

We also examined whether there was an association between dietary fish intake and the presence of any diabetic retinopathy (yes vs. no). In our study, dietary fish intake was associated with any diabetic retinopathy (yes vs. no) (odds ratio [OR], 0.35; 95% confidence interval (CI): 0.17 to 0.72; P = 0.004, adjusting for age, gender and race), comparing those in quartile 3 versus quartile 1. This association remained significant even in the fully adjusted model (OR, 0.31; 95% CI: 0.14 to 0.69; P = 0.004). However, there was no association between dietary fish intake and any DR when comparing quartile 4 to quartile 1.

Given that retinal vascular caliber was hypothesized as a potential mechanism underlying the association between dietary fish intake and DR, we next examined the association between retinal vascular caliber and DR. Retinal venular caliber was significantly related to severity of DR (β = 1.01; 95% CI, 1.01 to 1.02; P = 0.009, adjusting for age, gender and race) whereas retinal arteriolar caliber was not correlated to severity of DR (β = 1.00; 95% CI, 0.99 to 1.01; P = 0.974, adjusting for age, gender and race).

### Association of Fish intake with Retinal Vascular Caliber

Tables 4 and 5 show the association between fish intake and mean arteriolar and venular caliber by severity of DR, respectively. In the multivariable-adjusted model, compared to lowest quartile of fish intake, highest quartile of fish intake was associated with a significant 19.2% wider arteriolar (β = 22.27 µm, 95% CI: 12.64 to 31.90; P < 0.001; Tables 4) and 18.9% wider venular (β = 32.00 µm, 95% CI: 17.56 to 46.43; P < 0.001; Table 5) calibers, in the no DR group. However, this significant trend of widened retinal vascular calibers with increased dietary fish intake was not seen in either the minimal-moderate DR or severe DR or worse categories. This exception is noted in the minimal-moderate DR group, where fish intake was significantly associated with a 6.86% narrower venular caliber (β = −14.10 µm, 95% CI: −27.08 to −1.12; P = 0.034; Table 5).

**Table 4:** Association between fish intake and retinal arteriolar caliber by severity of DR in multivariable linear regression models.

Fish intake category	Model 1		Model 2		
	β (95% CI)	P value	β (95% CI)	P value	% change from reference
No DR					
Quartile 1 (Reference)	119.68 ± 6.66		116.30 ± 6.44	P-trend^*^ < 0.001	
Quartile 2	8.81 (0.15 to 17.47)	0.046	11.49 (3.12 to 19.86)	0.008	9.89%
Quartile 3	10.23 (−0.10 to 20.56)	0.052	14.59 (4.73 to 24.45)	0.004	12.55%
Quartile 4	16.26 (6.30 to 26.23)	0.002	22.27 (12.64 to 31.90)	< 0.001	19.17%
Minimal to Moderate DR					
Quartile 1 (Reference)	134.75 ± 5.94		133.95 ± 5.66	P-trend^*^ = 0.502	
Quartile 2	0.97 (−6.52 to 8.47)	0.798	3.13 (−4.13 to 10.38)	0.396	2.34%
Quartile 3	7.15 (−5.42 to 19.73)	0.262	7.84 (−4.30 to 19.98)	0.204	5.85%
Quartile 4	−2.16 (−10.70 to 6.39)	0.618	−2.59 (−10.82 to 5.65)	0.536	1.93%
Severe DR or worse					
Quartile 1 (Reference)	125.15 ± 9.02		125.49 ± 9.43	P-trend^*^ = 0.888	
Quartile 2	−1.90 (−14.26 to 10.46)	0.76	−1.85 (−14.97 to 11.28)	0.779	1.47%
Quartile 3	0.90 (−16.48 to 18.27)	0.918	−0.70 (−18.80 to 17.40)	0.939	0.56%
Quartile 4	1.82 (−14.62 to 18.27)	0.825	1.52 (−15.87 to 18.91)	0.862	1.21%

CI = confidence interval; DR = diabetic retinopathy.

*P-value to test for differences in trend between categories of fish intake (quartiles).

Model 1 adjusted for age, gender and race.

Model 2 adjusted for age, gender, race, and variables with P < 0.10 in model 1 were included (smoking, systolic blood pressure and HbA1c level).

**Table 5:** Association between fish intake and retinal venular caliber by severity of DR in multivariable linear regression models.

Fish intake category	Model 1		Model 2		
	β (95% CI)	P value	β (95% CI)	P value	% change from reference
No DR					
Quartile 1 (Reference)	173.95 ± 9.84		169.56 ± 9.62	P-trend^*^ < 0.001	
Quartile 2	12.43 (−0.33 to 25.19)	0.056	16.28 (3.84 to 28.72)	0.011	9.60%
Quartile 3	16.45 (1.23 to 31.67)	0.034	21.04 (6.23 to 35.85)	0.006	12.41%
Quartile 4	24.39 (9.70 to 39.08)	0.001	32.00 (17.56 to 46.43)	<0.001	18.87%
Minimal to Moderate DR					
Quartile 1 (Reference)	204.21 ± 9.54		205.44 ± 8.80	P-trend^*^ = 0.036	
Quartile 2	−3.31 (−15.32 to 8.70)	0.587	−2.08 (−13.35 to 9.18)	0.715	1.01%
Quartile 3	4.43 (−15.72 to 24.57)	0.665	1.76 (−17.08 to 20.60)	0.854	0.86%
Quartile 4	−7.98 (−21.68 to 5.71)	0.251	−14.10 (−27.08 to −1.12)	0.034	6.86%
Severe DR or worse					
Quartile 1 (Reference)	191.75 ± 12.18		193.64 ± 12.31	P-trend^*^ = 0.696	
Quartile 2	1.76 (−14.93 to 18.45)	0.834	1.36 (−15.84 to 18.55)	0.875	0.70%
Quartile 3	8.20 (−15.27 to 31.66)	0.488	5.34 (−18.53 to 29.21)	0.656	2.76%
Quartile 4	3.26 (−18.95 to 25.47)	0.771	2.97 (−19.62 to 25.55)	0.794	1.53%

CI = confidence interval; DR = diabetic retinopathy.

*P-value to test for differences in trend between categories of fish intake (quartiles).

Model 1 adjusted for age, gender and race.

Model 2 adjusted for age, gender, race, and variables with P < 0.10 in model 1 were included (BMI, systolic blood pressure, HbA1c and triglycerides levels).

Studies have previously demonstrated the association of hypertension and dyslipidemia with retinal vascular caliber, we next examined whether these associations are observed in the current study. Specifically, persons with hypertension tended to have narrower retinal arteriolar (β = −8.94; 95% CI, −13.01 to −4.80; P < 0.001, adjusting for age, gender and race) as well as venular (β = −7.42; 95% CI, −13.82 to −1.04; P = 0.023, adjusting for age, gender and race) caliber. There were no significant association between dyslipidemia and retinal arteriolar (β = −3.42; 95% CI, −7.76 to 0.91; P = 0.121, adjusting for age, gender and race) or venular (β = −3.89; 95% CI, −10.47 to 2.68; P = 0.245, adjusting for age, gender and race) caliber. For Tables 2–5, the associations between the relations of DR/vessel caliber to fish intake remained similar after substituting hypertension for systolic blood pressure (data not shown).

Tables 6 and 7 show the association between fish intake and mean arteriolar and venular caliber by DR laser treatment status, respectively. In the multivariable-adjusted model, compared to lowest quartile of fish intake, highest quartile of fish intake was associated with a significant 7.00% wider mean arteriolar caliber in the no DR laser treatment group (β = 8.85 µm, 95% CI: 2.77 to 14.94; P-trend = 0.010; Table 6). However, there was no significant trend of retinal arteriolar caliber with dietary fish intake in the DR laser treatment group. Similarly, there was no significant association between retinal venular caliber and fish intake (Table 7).

**Table 6:** Association between fish intake and retinal arteriolar caliber by DR treatment status in multivariable linear regression models.

Fish intake category	Model 1		Model 2		
	β (95% CI)	P value	β (95% CI)	P value	% change from reference
No laser treatment					
Quartile 1 (Reference)	127.48 ± 4.40		126.50 ± 4.30	P-trend^*^ = 0.010	
Quartile 2	5.70 (0.06 to 11.35)	0.048	6.50 (1.02 to 11.98)	0.020	5.14%
Quartile 3	4.30 (−3.12 to 11.72)	0.255	6.19 (−1.04 to 13.43)	0.093	4.90%
Quartile 4	7.49 (1.24 to 13.73)	0.019	8.85 (2.77 to 14.94)	0.005	7.00%
DR laser treatment					
Quartile 1 (Reference)	123.18 ± 9.96		133.95 ± 5.66	P-trend^*^ = 0.465	
Quartile 2	−0.73 (−13.92 to 12.45)	0.912	−1.37 (−15.63 to 12.89)	0.848	1.11%
Quartile 3	6.24 (−15.22 to 27.70)	0.563	3.48 (−19.24 to 26.20)	0.759	2.81%
Quartile 4	−11.40 (−34.52 to 11.72)	0.328	−14.74 (−40.48 to 11.00)	0.255	11.91%

CI = confidence interval; DR = diabetic retinopathy.

*P-value to test for differences in trend between categories of fish intake (quartiles).

Model 1 adjusted for age, gender and race.

Model 2 adjusted for age, gender, race, and variables with P < 0.10 in model 1 were included (smoking, systolic blood pressure and HbA1c level).

**Table 7:** Association between fish intake and retinal venular caliber by DR treatment status in multivariable linear regression models.

Fish intake category	Model 1		Model 2		% change from reference
	β (95% CI)	P value	β (95% CI)	P value	
No laser treatment					
Quartile 1 (Reference)	189.89 ± 6.86		189.49 ± 6.76	P-trend^*^ = 0.157	
Quartile 2	5.51 (−3.31 to 14.33)	0.220	6.40 (−2.24 to 15.02)	0.146	3.37%
Quartile 3	4.42 (−7.19 to 16.02)	0.454	4.41 (−6.99 to 15.81)	0.447	2.33%
Quartile 4	8.31 (−1.46 to 18.07)	0.095	8.05 (−1.57 to 17.67)	0.101	4.25%
DR laser treatment					
Quartile 1 (Reference)	189.02 ± 13.38		189.46 ± 13.94	P-trend^*^ = 0.683	
Quartile 2	2.79 (−14.91 to 20.49)	0.753	4.21 (−14.65 to 23.07)	0.655	1.11%
Quartile 3	12.19 (−16.61 to 41.00)	0.400	9.03 (−20.25 to 38.31)	0.538	2.81%
Quartile 4	−4.99 (−36.03to 26.04)	0.748	1.43 (−32.48 to 35.34)	0.933	11.91%

CI = confidence interval; DR = diabetic retinopathy.

*P-value to test for differences in trend between categories of fish intake (quartiles).

Model 1 adjusted for age, gender and race.

Model 2 adjusted for age, gender, race, and variables with P < 0.10 in model 1 were included (BMI, systolic blood pressure, HbA1c and triglycerides levels).

## Discussion

In this clinical sample of multiethnic Asian adults with type 2 diabetes living in Singapore, greater fish consumption was associated with a decreased likelihood of having severe DR or worse. In addition, greater fish consumption was associated with wider retinal vascular caliber in subjects without retinopathy. Whether these associations are causal remain to be seen. To the best of our knowledge this is the first study to show this association in an Asian population.

Our study showed that regular consumption of fish was significantly associated with reduced odds of having severe DR or worse. Therefore, our results concur with findings from Western populations that fish consumption may protect against VTDR. A recent large scale prospective study reported that Spanish adults with type 2 diabetes, who consumed at least two servings of oily fish per week had a significantly decreased risk of incident VTDR^5^. The reproducibility of such an association of fish intake and DR in another ethnic group i.e. Asians, suggests that a biological association for fish intake and DR risk may be broadly consistent across ethnic boundaries. Because diet modification is the cornerstone of diabetes care, such a finding will be critical and useful in both clinical and research settings.

Our measurement of vascular parameters may shed some insight into the mechanisms underlying the effects of fish intake. Microvascular complications have been implicated in the processes that lead to early DR^25–28^. There is some controversy whether early diabetes is associated with increased or decreased retinal blood flow^8,29–34^ but in the late stages of the disease, there is a pronounced decrease in retinal blood perfusion^35^. Previous studies have shown that narrower retinal arteriolar caliber is associated with increased risk of diabetes, DR and other microvascular diabetic complications such as diabetic nephropathy, coronary microvascular disease and lacunar stroke^36–39^. Thus, the association of increased fish intake with wider retinal arteriolar caliber is consistent with the protective effect of fish intake on DR. However, our finding that higher fish intake was related to wider venular caliber is more difficult to explain, because wider venular caliber has been previously associated with increased risk of DR^40^.

Of noteworthy, there was a lack of an association between fish intake and vascular parameters among DR patients. This may have been due to the small fraction of the DR study population (minimal-moderate DR group: 20%; severe DR or worse group: 20%). Another explanation for the discordant results seen within the DR group pertains to the effects of higher blood pressure, which may diminish the effect of fish consumption on vascular parameters. Studies have reported that the independent effect of elevated blood pressure on retinal arteriolar narrowing and venular widening^41,42^. Thus, the extent to which high blood pressure on vascular parameters may mask the effects of fish intake requires further study.

Protective cardiovascular effects of fish intake are believed to be partially due to omega-3 fatty acids^43^. A growing body of evidence from cardiovascular disease studies indicates that the anti-inflammatory properties of omega-3 free fatty acids is associated with a decrease in plasma concentrations of pro-inflammatory cytokines such as tumor necrosis factor, interleukin 1 beta, and interleukin 6^44^. The reason why retinal vascular caliber is increased in diabetes is not known, but increased C-reactive protein is associated with wider venular diameter in several populations^45–47^. As such, one would hypothesize that an anti-inflammatory effect of omega-3 fatty acids would lead to smaller retinal calibers. Our data therefore more likely indicate that omega-3 fatty acids promote their effects via vasodilatory effects. Indeed, it has been shown that fish oil increased aortic endothelial nitric oxide synthase expression and nitric oxide production in atherosclerosis-prone apolipoprotein E (−/−) mice^48^. In addition, eicosapentaenoic acid improved vascular endothelial function in a type 2 model of diabetes in rats by normalizing the balance between vasodilator and vasoconstrictor mediators^49^. This is compatible with eicosapentaenoic acid reducing endothelin-1 production in cultured human endothelial cells^50^. Both nitric oxide and endothelin are important regulators of retinal vascular tone^51–55^ and a shift in endothelial derived vasoactive substance may therefore well explain the dependence of retinal vascular caliber on fish intake.

The strengths of this study include standardized grading protocols to define DR by trained graders and a comprehensive clinical examination. Our results should be interpreted cautiously. First, the available evidence is based on a cross-sectional study design, with known limitations of inferring temporal associations. It is also possible that clinical diagnosis of DR could lead to greater dietary fish intake in diabetics, for fear of imminent sight loss. Longitudinal studies are warranted to confirm the protective role of fish intake in the pathogenesis of DR and our results may also inform future intervention trials to determine whether fish intake can be truly beneficial for individuals with type 2 diabetes. Second, we used the FFQ to assess dietary intake, which can be subjected to recall bias and misreporting. Third, our FFQ did not capture specific intake of oily fish, key source of omega-3 fatty acids. Therefore, it is unclear if omega-3 fatty acids drive the favorable association between fish and DR. Future studies can utilize objective plasma nutrient biomarker to measure omega-3 fatty acids content to further elucidate the inter-relationship between dietary choices and risk of future DR. Fourth, it is of concern that the presumed effects of fish-derived omega-3 may be masked by unhealthy fats, in particular relating to those fish items consumed along with unhealthy fats. In particular, cooking methods such as deep frying and fish cooked in coconut curry may potentially add unhealthy fats during the cooking process (i.e. saturated and trans fat), which may counter-balance the health benefits of fish intake. The Australian Diabetes Management Project, our sister study conducted in Australia, found that increasing saturated fatty acid intake was associated with an increased likelihood of the presence and severity of DR^4^. The most common cooking method for fish consumed by our participants is steaming (51.1%), followed by stir-fried/pan-fried/deep fried (25.5%), deep fried with batter (10.2%), curry without coconut (4.7%), assam pedas (4.1%), curry with coconut (2.8%), and grilled (1.6%). Only 13.0% of participants reported intake of fish deep fried with batter or fish cooked in coconut curry, whereas majority still consumed mainly fish prepared with healthier cooking methods. Thus, the distribution of fish cooked in unhealthy fat was too narrow in this population to be evaluated as a detailed exposure. Fifth, we did not measure the participants’ serum omega 3 levels, which may provide a direct biological explanation to the associations with severity of DR and retinal vascular caliber. This was because fasting blood samples were not obtained from the participants. Their non-fasting lipid profile may be affected by their dietary intake prior to the visit. Sixth, we saw a lack of significant finding of DR with the fourth quartile of fish intake. This is unclear despite the P-trend showing significance for the ordered relationship across the categories of a fish intake and DR. In addition, the protective effect of fish intake on DR can also be seen when fish intake was considered as a continuous variable. Last, because of the clinical sample, our results may not be generalizable to the general population. However, our results are relevant to the clinic population, which has a high proportion of cases with DR.

Consistent with the other studies on several ethnic groups, greater fish consumption among type 2 diabetes was found to be associated with lower risk of DR in Asian persons in this study. In addition, greater fish intake was also associated with wider retinal vascular caliber among diabetics without any signs of retinopathy. It remains unclear whether the relationship between dietary fish intake with retinal microvascular and DR is a result of a cause or consequence of, but our findings suggest possible links via postulated mechanisms of inflammation. Our data indicate a complex relationship among dietary fish intake, retinal vascular caliber, and severity of DR. Future studies are required to draw more definitive inferences on the direction of causality.
